# The Immediate Intramedullary Nailing Surgery Increased the Mitochondrial DNA Release That Aggravated Systemic Inflammatory Response and Lung Injury Induced by Elderly Hip Fracture

**DOI:** 10.1155/2015/587378

**Published:** 2015-07-27

**Authors:** Li Gan, Jianfeng Zhong, Ruhui Zhang, Tiansheng Sun, Qi Li, Xiaobin Chen, Jianzheng Zhang

**Affiliations:** ^1^Department of Orthopaedics, Zhujiang Hospital, Southern Medical University, Guangzhou, Guangdong 510282, China; ^2^Department of Orthopaedics, Beijing Army General Hospital, Beijing 100700, China; ^3^Department of Respiratory Diseases, Chronic Airways Diseases Laboratory, Nanfang Hospital, Southern Medical University, Guangzhou, Guangdong 510282, China

## Abstract

Conventional concept suggests that immediate surgery is the optimal choice for elderly hip fracture patients; however, few studies focus on the adverse effect of immediate surgery. This study aims to examine the adverse effect of immediate surgery, as well as to explore the meaning of mtDNA release after trauma. In the experiment, elderly rats, respectively, received hip fracture operations or hip fracture plus intramedullary nail surgery. After fracture operations, the serum mtDNA levels as well as the related indicators of systemic inflammatory response and lung injury significantly increased in the rats. After immediate surgery, the above variables were further increased. The serum mtDNA levels were significantly related with the serum cytokine (TNF-*α* and IL-10) levels and pulmonary histological score. In order to identify the meaning of mtDNA release following hip fracture, the elderly rats received injections with mtDNA. After treatment, the related indicators of systemic inflammatory response and lung injury significantly increased in the rats. These results demonstrated that the immediate surgery increased the mtDNA release that could aggravate systemic inflammatory response and lung injury induced by elderly hip fracture; serum mtDNA might serve as a potential biomarker of systemic inflammatory response and lung injury following elderly hip fracture.

## 1. Introduction

Hip fracture is the most serious consequence of falling in the elderly; 87% to 96% of hip fracture patients are 65 years of age or older, and hip fractures are associated with numerous complications and increased mortality [1, 2]. The overall in-hospital mortality of elderly patients with hip fracture is nearly 5.0% [3]. The major causes of the high mortality are complications associated with the fracture event [4]. Of these complications, lung infection is the most common cause of mortality [1]. Traditionally, long-term bed rest is considered the main risk factor for the development of lung infections and death, and early and ultraearly surgery in hip fracture patients can reduce the postoperative complications and mortality associated with long-term best rest [5–8]. However, our recent studies have demonstrated that hip fracture in the elderly can induce systemic inflammatory responses and lung injury, which increase the risk of pulmonary infection and death during the postinjury period [9–11]. In addition, damage control orthopedics (DCO) findings suggest that immediate definitive fixation of long-bone fractures can be detrimental to patients who are physiologically unstable [12, 13]. Therefore, it is necessary to confirm the influence of immediate surgery on systemic inflammatory responses and lung injury after hip fracture in elderly patients.

Mitochondria are evolutionary endosymbionts that were derived from bacteria [14] and therefore contain bacterial molecular motifs. Previous studies have implicated mitochondrial damage-associated molecular patterns (DAMPs) with sterile inflammation [15–18]. In addition, mtDNA has been detected in the plasma of trauma patients and causes functionally important immune consequences [19–21]. However, the role of mtDNA remains uncertain in systemic inflammatory responses and lung injury induced by hip fracture in the elderly. Our study aimed to investigate the patterns of mtDNA release in a fracture model and a fracture plus surgery model as well as to evaluate the relationship between mtDNA and posttraumatic inflammation. Understanding the relationship between mtDNA and posttraumatic inflammation may help elucidate the underlying pathophysiological mechanisms through which trauma induces SIRS and may facilitate the identification of novel therapeutic targets. In addition, understanding the patterns of mtDNA release may help to predict the occurrence of posttraumatic inflammation.

## 2. Materials and Methods

This paper consisted of two parts. In the first part, we studied the influence of immediate surgery on the elderly hip fracture rats. In the second part, we investigated the effects of serum mtDNA on the elderly rats.

### 2.1. Animals

Twenty- to 23-month-old rats are considered elderly [22]. A total of 85 elderly male Sprague-Dawley (SD) rats (age: 22-23 months; weight: 450–550 g) were purchased from Beijing Haiwang Experimental Animal Center and were housed in a climate-controlled barrier facility with 12 h light/dark cycles at 24 ± 2°C and free access to food and water for a period of at least 1 week prior to the experimental procedures; the rats were then maintained at the Beijing Military Generational Hospital. The experiments were performed according to the guidelines for Experimental Animal Care and Use approved by the Beijing Military General Hospital Ethics Committee (Permit number: 20140218).

### 2.2. Grouping of Animals

In order to study the influence of immediate surgery on the elderly hip fracture rats, in the first part of the experiment, 60 elderly rats were randomly divided into three groups. The sham group (*n* = 20) only received anesthesia, the fracture group (*n* = 20) received anesthesia and underwent hip fracture operations, and the fracture plus surgery group (*n* = 20) underwent proximal femoral intramedullary nail surgery in addition to receiving anesthesia and undergoing hip fracture operations.

### 2.3. Fracture Model

The rats in the fracture group and the fracture plus surgery group were anesthetized with 10% chloral hydrate (3.5 mL kg^−1^, i.p.) and then placed in a prone position on the base of a blunt guillotine ramming apparatus. Rats were fixed after the proximal femur was identified and marked. The 500 g blunt guillotine was lifted to a 15 cm height and was allowed to fall freely along the axis. The force of the falling object resulted in a unilateral closed proximal femoral fracture (hip fracture).

### 2.4. Proximal Femoral Intramedullary Pinning Surgery

The surgery was performed immediately after fracture according to Sears et al. [23]. Prophylactic preoperative antibiotics (gentamicin, 5 mg/kg) were administered before incision. A proximal lateral femoral incision (approximately 0.5 cm) was made, and the fracture ends were exposed. A 1.25 mm Kirschner wire was retrogradely inserted into the proximal femoral medullary cavity using a drill, and the proximal part of the pin emerged from the piriform fossa. The proximal portion of the pin was connected to the drill, and the distal femoral medullary cavity was entered. Fracture reduction was performed during the insertion of the Kirschner wire. The proximal portion of the pin was cut, and the incision was closed with 3-0 polyglycolic acid sutures. The animals in the fracture group and the fracture plus surgery group were administered Buprenex (buprenorphine, 0.1 mg/kg) every 10–12 h for pain control.

### 2.5. Collection of Samples

The rats were sacrificed at 8, 24, 48, and 72 h after treatment. Each thorax was opened rapidly; subsequently, blood samples were collected by heart puncture and were centrifuged at 3000 rpm for 10 min to separate the serum from cellular blood components. The serum was stored at −80°C for the later mtDNA and cytokine assays. Lung tissues were quickly harvested. The left trachea was exposed, and the bronchoalveolar lavage fluid was collected three times through a tracheal cannula with autoclaved PBS that was instilled up to a total volume of 1.0 mL and then centrifuged at 3000 rpm for 10 min. The supernatant was frozen at −80°C until the subsequent cytokine and protein assays. The right middle lung lobes were removed for the MPO and NE assays. The rest of the right lung was fixed immediately in 10% formalin and stored at 4°C for subsequent histological observation and pathological scoring.

### 2.6. Histological Analysis

Lung specimens were embedded in paraffin. Tissue sections (5–8 *μ*m) were prepared and stained with hematoxylin and eosin (H&E). Briefly, 3 slices were randomly selected from each rat, and 3 fields of each slice were reviewed under a microscope (100x magnification, Olympus DP71, Tokyo, Japan). All slides were examined and scored by an experienced pathologist (Ru Hui) who was blinded to the experimental groups based on the lung injury scoring system [24]. The score was based on categories of inflammatory cell infiltration, pulmonary edema, congestion, and intra-alveolar hemorrhage that were graded on a scale of normal (0), mild (1), moderate (2), or severe (3) injury, with a maximum possible score of 12.

### 2.7. Protein Assay

The total protein concentration in the BALF was determined using a BCA protein assay kit (Pierce Biotechnology, USA) following the manufacturer's instructions.

### 2.8. Cytokines Assay

The cytokines (TNF-*α* and IL-10) in the serum and BALF were measured using an enzyme-linked immunosorbent assay (ELISA) system (R&D Systems, USA) following the manufacturer's instructions.

### 2.9. MPO and NE Activity Assay

The right middle lung lobes were placed in 1 mL of homogenization buffer (4°C). Samples were homogenized and incubated at 4°C for 1 h. The final homogenate was centrifuged at 10,000 rpm for 15 min. Tissue supernatants were used for the determination of MPO and NE activity. MPO activity was measured using an MPO kit (Miltenyi Biotec, Germany) according to the manufacturer's instructions. NE activity was determined using a quantitative sandwich enzyme immunoassay (R&D Systems, USA) following the manufacturer's instructions.

### 2.10. Serum mtDNA Isolation

Serum was extracted before the blood samples were incubated at 37°C for 1 h and centrifuged at 2500 rpm for 10 min. Serum DNA was extracted from 200 *μ*L of serum using a QIAamp Blood Mini Kit based on affinity columns (Qiagen, Hilden, Germany) according to the manufacturer's recommendations.

### 2.11. FQ-PCR

MtDNA gene primers were designed according to a previous study [25]: forward, CAGCCGCTATTAAAGGTTCG; and reverse, CCTGGATTACTCCGGTCT GA. The product size was 79 bp. The mtDNA plasmid was constructed using a TA cloning kit as follows: the purified PCR products were linked into the pMD18-T vector (TaKaRa, Japan), and the connective product was transformed to DH5*α* competent* Escherichia coli*. The positive* E. coli* clones were screened and enriched, and then mtDNA was extracted from the plasmid and measured. A standard curve was generated by six dilutions of DNA (rang 10^2^–10^7^ copies/*μ*L) using an ABI 7500 sequence detection system (ABI, USA). FQ-PCR reactions were conducted in 96-well plates within a total volume of 20 *μ*L/well containing the following reagents: SYBR Premix Ex Taq II (2x): 10 *μ*L; forward primer: 0.8 *μ*L; reverse primer: 0.8 *μ*L; ROX reference Dye II (50x): 0.4 *μ*L; DNA: 2 *μ*L; and ddH_2_O: 6 *μ*L. The PCR kit was purchased from TaKaRa Company (TaKaRa, Japan). The PCR reaction was conducted using the following conditions: 95°C for 30 s, followed by 95°C for 5 s and 60°C for 34 s, repeated for 40 cycles. Each sample and DNA standard was analyzed in duplicate, and the mean value was used for quantification. Only standard curves with a coefficient of correlation >0.96 were accepted.

### 2.12. Rat Femur mtDNA Preparation

In order to identify the effects of serum mtDNA on the elderly rats, in the second part of the experiment, 5 elderly rats were sacrificed by cervical dislocation, their femurs were collected, and the femoral mitochondria was isolated using a mitochondrial isolation kit (Pierce, USA) according to the manufacturer's instructions. Subsequently, the mitochondrial pellets were resuspended in Hanks' balanced salt solution (HBSS) (Gibco Life Technologies, USA). After a protease inhibitor cocktail (1 : 100) (Qiagen, USA) was added, the suspension was sonicated on ice (VCX130-Vibra Cell, USA) at 100% amplitude 10 times for 30 s each with 30 s intervals. The mtDNA was isolated by centrifugation at 15,000 ×g for 10 min at 4°C followed by centrifugation at 100,000 ×g at 4°C for 30 min. The mtDNA were extracted from the isolated mitochondrial pellets using the DNeasy Blood and Tissue Kit (Qiagen) following manufacturer's instructions. The purity and concentrations of the mtDNA were determined by FQ-PCR and spectrophotometry, respectively.

### 2.13. mtDNA Inoculation of Animals

20 elderly rats were randomly divided into control group and mtDNA group. The control group (*n* = 10) received intravenous injections with 1 mL PBS; the mtDNA group (*n* = 10) received injections with 1 mL of 10 *μ*g/mL mtDNA. The mtDNA concentration was selected according to our previous experiments [21]. After 24 h, the animals were sacrificed by cervical dislocation, and then specimens were collected and detected as the first part.

### 2.14. Statistical Analysis

Data are presented as the means ± standard deviation. All data were analyzed by SPSS software (version 13.0). One-way ANOVA with Bonferroni post hoc tests were performed to compare the data from all groups at each monitoring point. Quantitative data were compared between two groups using the *t*-test. To evaluate the predictive value of mtDNA and cytokines (TNF-*α* and IL-10), a linear regression analysis was applied to measure the relationships between pulmonary histological score and the mtDNA and cytokine levels. A *P* value of less than 0.05 was considered statistically significant.

## 3. Results

### 3.1. The Influence of Immediate Surgery on Elderly Hip Fracture Rats

#### 3.1.1. Changes of mtDNA, TNF-*α*, and IL-10 Levels in Serum

**Figure 1:**
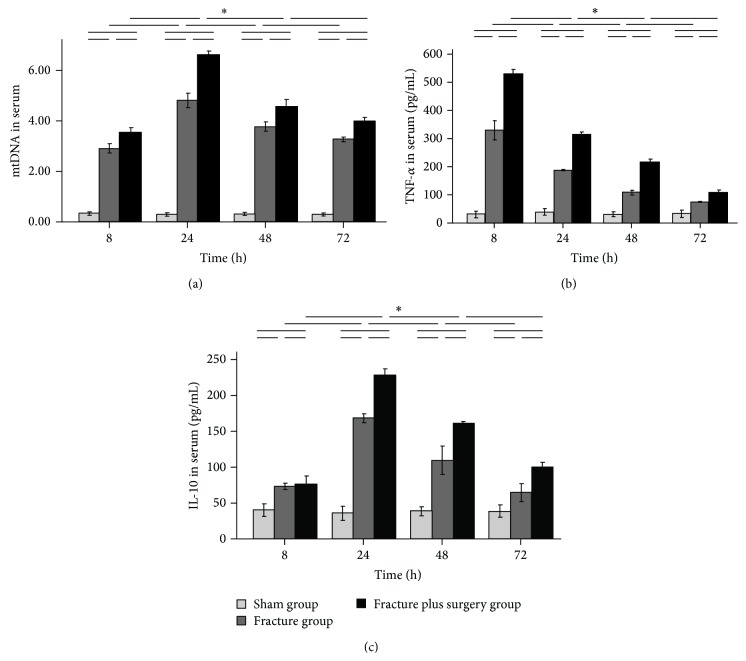
Time course of serum mtDNA (a), TNF-*α* (b), and IL-10 (c) levels. Results are expressed as mean ± standard error. ^∗^ *P* < 0.05 when compared to other groups.

The serum mtDNA, TNF-*α*, and IL-10 levels in the fracture group and the fracture plus surgery group increased rapidly after treatment, peaked at 24 h (with the exception of TNF-*α*, which peaked at 8 h), and were significantly higher than those in the sham group at all time points (all *P* < 0.05). Compared with the fracture group, at 8, 24, 48, and 72 h after treatment, the above variables were higher in the fracture plus surgery group (all *P* < 0.05) (Figure 1).

#### 3.1.2. Pulmonary Changes

**Figure 2:**
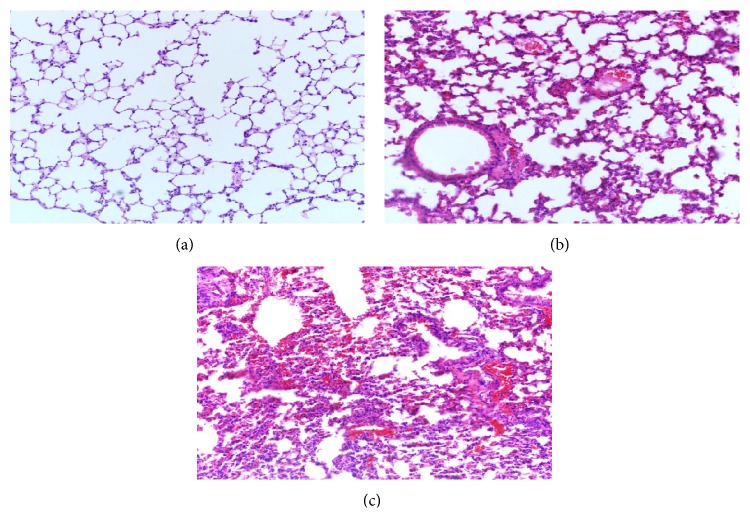
Representative H&E sections of pulmonary tissues (magnification, 100x). After treatment, the sham group (a) had no obvious inflammation; the fracture group (b) and the fracture plus surgery group (c) showed typical symptoms of acute lung injury.

As shown in Figure 2(a), the lung tissue structure of the sham group was clear, and the alveolar walls were normal without significant inflammatory corpuscle infiltration after treatment. However, the pulmonary tissue slices of the fracture group (Figure 2(b)) and the fracture plus surgery group (Figure 2(c)) exhibited increased congestion, pulmonary edema, polymorphonuclear and mononuclear cell infiltrates, and damaged alveolar architecture.

**Figure 3:**
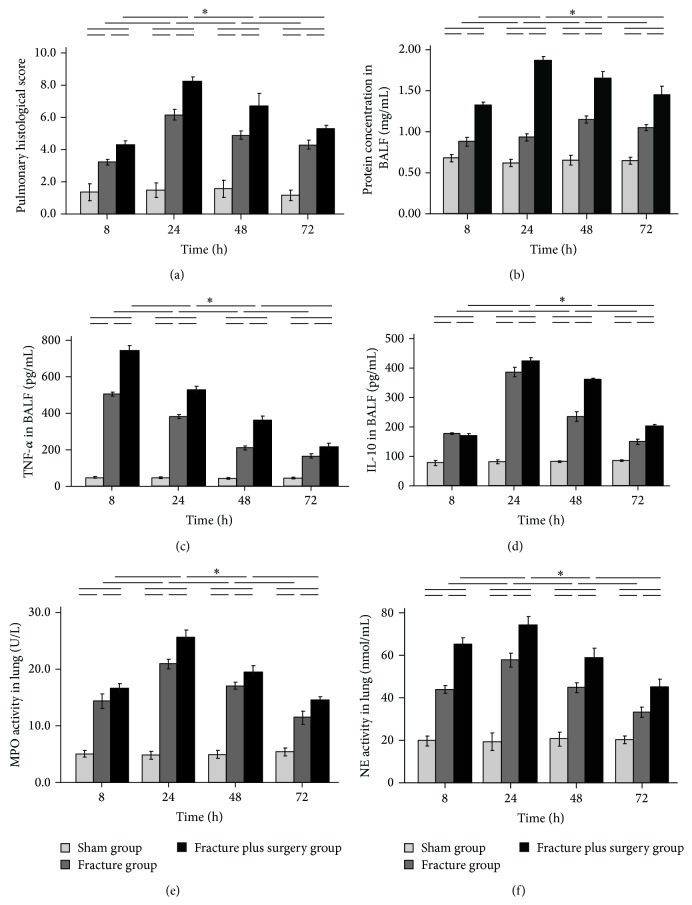
Time course of the pulmonary histological score (a), the protein (b), TNF-*α* (c), and IL-10 (d) concentrations in BALF and the lung tissue MPO (e) and NE (f) activity. Results are expressed as mean ± standard error. ^∗^ *P* < 0.05 when compared to other groups.

As shown in Figure 3, at 8, 24, 48, and 72 h after treatment, the pulmonary histological score, the cytokine (TNF-*α* and IL-10) and protein concentrations in BALF, and the lung tissue MPO and NE activity of the fracture group and the fracture plus surgery group increased rapidly after treatment, peaked at 24 h (except TNF-*α*, which peaked at 8 h), and were higher than the respective values in the sham group at all time points (all *P* < 0.05). Compared with the fracture group, at 8, 24, 48, and 72 h after treatment, the above variables were higher in the fracture plus surgery group (all *P* < 0.05).

### 3.2. The Results of the Linear Regression Analysis

**Figure 4:**
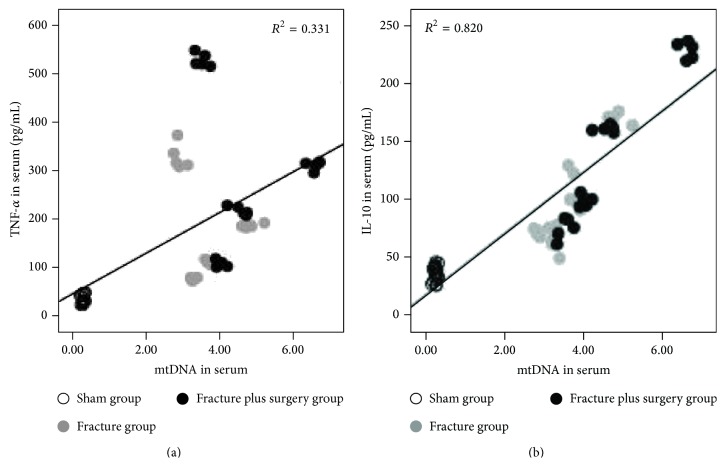
Scatter plots and regression lines showed that the serum TNF-*α* (a) and IL-10 (b) concentrations exhibited a significant relationship with the serum mtDNA levels.

**Figure 5:**
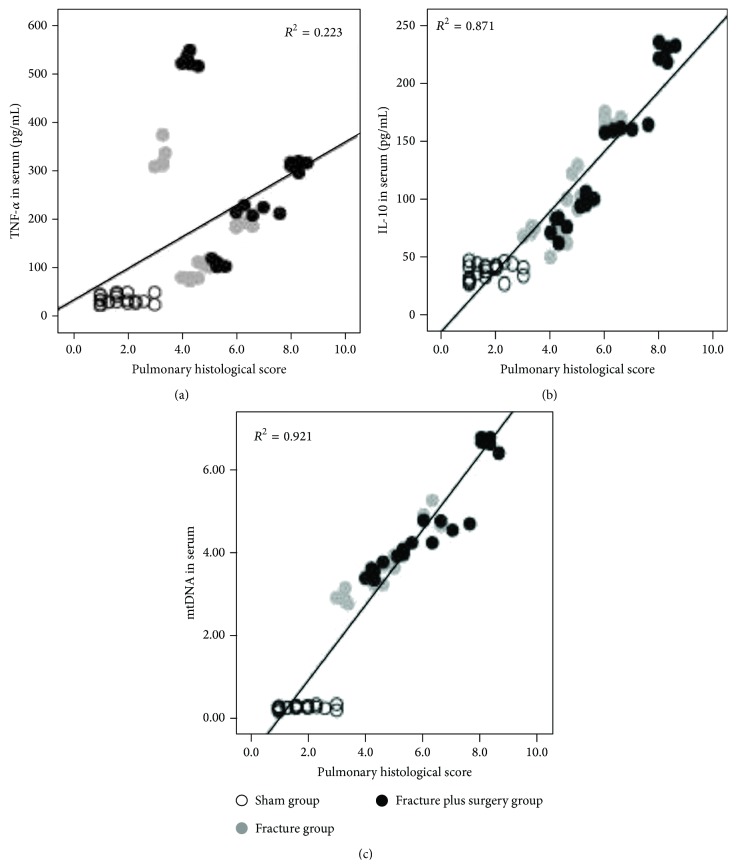
Scatter plots and regression lines showed that the serum TNF-*α* (a) level was not significantly correlated with the pulmonary histological score; however, the serum IL-10 (b) and mtDNA (c) levels exhibited a significant relationship with the pulmonary histological score.

The results of the linear regression analysis revealed that the serum mtDNA levels exhibited a significant relationship with the serum TNF-*α* (*B* = 0.004, *P* = 0.000) and IL-10 (*B* = 0.025, *P* = 0.000) concentrations (Figure 4), and the serum mtDNA (*B* = 0.863, *P* = 0.000) and IL-10 (*B* = 0.010, *P* = 0.001) levels were significantly correlated with the pulmonary histological score (serum TNF-*α* levels were removed; *P* = 0.180) (Figure 5).

### 3.3. The Effects of Serum mtDNA on the Elderly Rats

**Figure 6:**
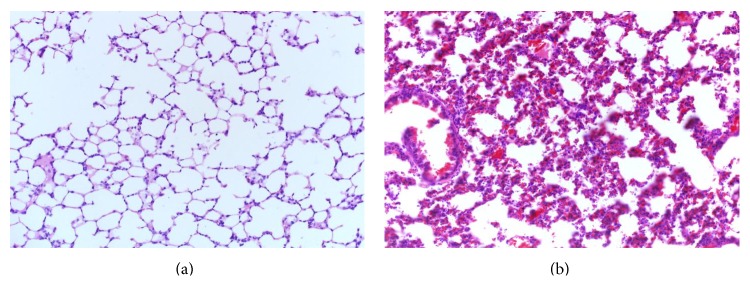
Representative H&E sections of pulmonary tissues (magnification, 100x). After treatment, the control group (a) had no obvious inflammation and the mtDNA (b) showed typical symptoms of acute lung injury.

**Figure 7:**
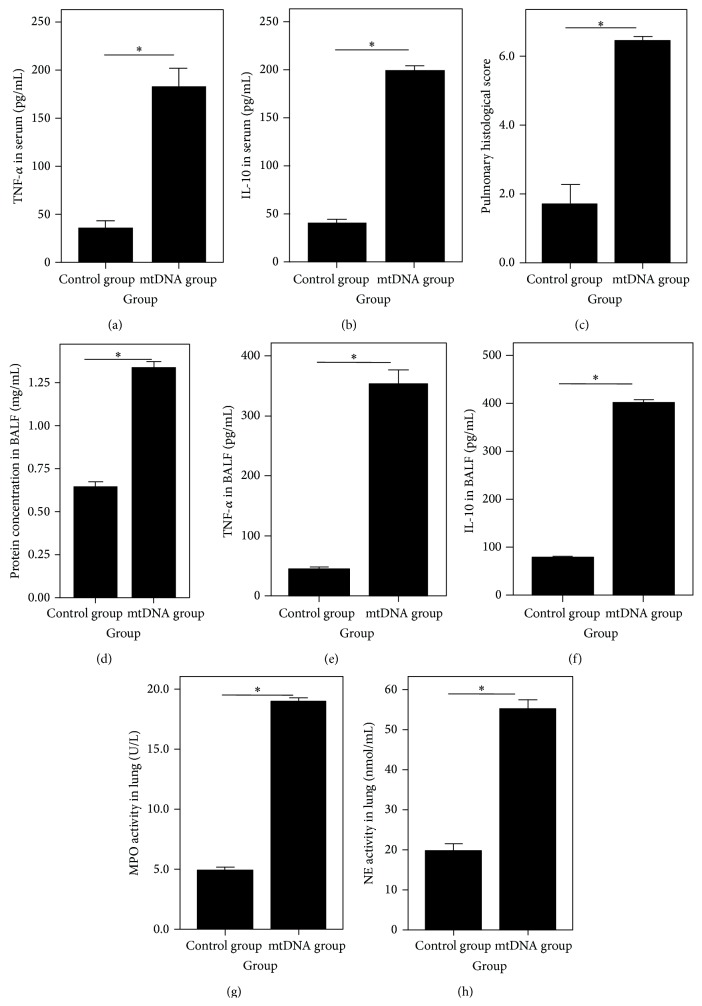
The serum TNF-*α* (a) and IL-10 (b) levels, the pulmonary histological score (c), the protein (d), TNF-*α* (e) and IL-10 (f) concentrations in BALF, and the lung tissue MPO (g) and NE (h) activity. Results are expressed as mean ± standard error. ^∗^ *P* < 0.05 when compared to other groups.

As shown in Figure 6(a), the lung tissue structure of the control group had no obvious manifestation of inflammation. However, the pulmonary tissue slices of the mtDNA group (Figure 6(b)) showed typical manifestations of acute lung injury. At 24 h after treatment, the serum TNF-*α* (Figure 7(a)) and IL-10 (Figure 7(b)) levels, the pulmonary histological score (Figure 7(c)), the protein (Figure 7(d)), TNF-*α* (Figure 7(e)), and IL-10 (Figure 7(f)) concentrations in BALF, and the lung tissue MPO (Figure 7(g)) and NE (Figure 7(h)) activity in the mtDNA group were significantly higher than the respective values in the control group (all *P* < 0.05).

## 4. Discussion

There is indisputable evidence that trauma can trigger an inflammatory response [26]. However, not all traumas result in an uncontrolled systemic inflammatory response. When trauma induces a host defense response, which is defined as systemic inflammatory response syndrome (SIRS) and is characterized by local and systemic release of proinflammatory cytokines, anti-inflammatory mediators are produced (compensatory anti-inflammatory response syndrome, CARS) to inhibit SIRS. Only when the organisms are overwhelmed by trauma is the balance broken, and an imbalance in the immune responses leads to the development of multiorgan failure (MOF) [27–30]. The lungs are the first and primary target organ to be affected in the postinjury period, and lung injury caused by posttraumatic inflammatory reactions increases mortality risk [23, 31, 32]. Recently, a number of studies have reported that the elderly possess a reduced physiological reserve and are more vulnerable to posttraumatic inflammatory reactions; even minor trauma can cause severe autodestructive proinflammatory cytokine release that is uncompensated by anti-inflammatory mediators [33–35]. This is consistent with our experimental results. In our experiment, compared with the sham group, at 8, 24, 48, and 72 h after treatment, the serum TNF-*α* and IL-10 levels, the pulmonary histological score, the cytokine (TNF-*α* and IL-10) and protein concentrations in the BALF, and the lung tissue MPO and NE activities were significantly increased in the fracture group. These findings suggest that hip fracture resulted in significant systemic inflammation and lung injury in the elderly rats.

Elderly trauma patients require special treatment because of their higher mortality rate following trauma, even minor trauma [36–38]. Surgical and clinical dogma states that if, at all possible, any patient with a hip fracture should undergo surgical fixation as soon as possible because any delay in surgery is thought to increase the risk of complications and death [6, 7, 39, 40]. However, hip fracture in the elderly can induce systemic inflammatory responses and lung injury, which increase the risk of pulmonary infection and death during the postinjury period [9–11]. In addition, the DCO proposes that immediate definitive fixation of long-bone fractures can be detrimental to patients who are physiologically unstable [12, 13]. Therefore, the adverse effects of immediate surgery are worth investigating. In our experiment, compared with the fracture group, the serum TNF-*α* and IL-10 levels, the pulmonary histological score, the cytokine (TNF-*α* and IL-10) and protein concentrations in the BALF, and the lung tissue MPO and NE activity were significantly increased in the fracture plus surgery group. These findings indicate that immediate intramedullary nailing surgery aggravated the systemic inflammatory reaction and lung injury induced by elderly hip fracture.

Previous studies have reported that elderly hip fracture induces a systemic inflammatory response and lung injury and increases the incidence of complications and mortality in elderly patients [1, 9, 11, 30, 32, 35]. Any neglect or misdiagnosis of this condition may produce disastrous results. Early diagnosis and evaluation of the systemic inflammatory response and lung injury after trauma are crucial for timely correction of this complicated syndrome. Potential biomarkers include acute phase proteins, cytokines, and chemokines [41–44]; however, none of these markers are sufficiently specific due to overlap with other inflammatory diseases [45–47]. Ideal biomarkers with higher sensitivity and specificity remain to be identified.

Recent studies have demonstrated that mtDNA is a critical activator of inflammation and the innate immune system. The release of mtDNA by cellular injury is a key link between trauma, inflammation, and SIRS [20, 48, 49]. A previous study reported that mtDNA from femoral reamings activated inflammation [18]. Our previous study also indicated that mtDNA induces a systemic inflammatory response and lung injury [21]. Therefore, we hypothesized that circulating mtDNA levels are associated with the systemic inflammatory response and lung injury induced by elderly hip fracture and might serve as a viable biomarker for the inflammatory response.

In the first part of our study, at 8, 24, 48, and 72 h after treatment, compared with the sham group, the serum mtDNA levels were significantly increased in the fracture group and the fracture plus surgery group; the serum mtDNA levels of the fracture plus surgery group were higher than those in the fracture group. It indicated that hip fracture releases mtDNA into the circulation; the immediate intramedullary nailing surgery increases the release of mtDNA. In addition, the results of the linear regression analysis revealed that the serum mtDNA levels were significantly correlated with serum cytokine (TNF-*α* and IL-10) levels and the pulmonary histological score. Compared with serum cytokines (TNF-*α* and IL-10), serum mtDNA was more closely correlated with the pulmonary histological score, indicating that the increasing release of mtDNA may be the reason of immediate intramedullary nailing surgery aggravating the systemic inflammatory response and lung injury induced by hip fracture in the elderly.

In order to identify the effects of serum mtDNA on the rats, the second part of our study was carried out. The result showed that the serum TNF-*α* and IL-10 levels, the pulmonary histological score, the cytokine (TNF-*α* and IL-10) and protein concentrations in the BALF, and the lung tissue MPO and NE activity were significantly increased in the rats which received mtDNA injections. It indicated that the mtDNA could cause systemic inflammation and lung injury in the elderly rats.

In general, hip fracture induces substantial mtDNA release, systemic inflammatory response, and lung injury in the elderly. Immediate intramedullary nailing surgery could aggravate the above pathological states. Serum mtDNA exhibited a significant relationship with posttraumatic inflammation and can serve as a potential biomarker of the systemic inflammatory response and lung injury following hip fracture in the elderly. Further study will define the value of serum mtDNA in the early diagnosis and prognosis of posttraumatic inflammation and may aid in the evaluation of the physical condition of trauma patients and inform the proper surgical approach.
